# Sex Allocation in a Polyembryonic Parasitoid with Female Soldiers: An Evolutionary Simulation and an Experimental Test

**DOI:** 10.1371/journal.pone.0064780

**Published:** 2013-06-03

**Authors:** Max Bügler, Polychronis Rempoulakis, Roei Shacham, Tamar Keasar, Frank Thuijsman

**Affiliations:** 1 Chair of Computational Modeling and Simulation, Technische Universität München, Munich, Germany; 2 Biology and Environment, University of Haifa, Tivon, Israel; 3 Evolutionary and Environmental Biology, University of Haifa, Haifa, Israel; 4 Knowledge Engineering, Maastricht University, Maastricht, The Netherlands; CNRS, Université de Bourgogne, France

## Abstract

Parasitoid wasps are convenient subjects for testing sex allocation theory. However, their intricate life histories are often insufficiently captured in simple analytical models. In the polyembryonic wasp *Copidosoma koehleri*, a clone of genetically identical offspring develops from each egg. Male clones contain fewer individuals than female clones. Some female larvae develop into soldiers that kill within-host competitors, while males do not form soldiers. These features complicate the prediction of *Copidosoma*’s sex allocation. We developed an individual-based simulation model, where numerous random starting strategies compete and recombine until a single stable sex allocation evolves. Life-history parameter values (e.g., fecundity, clone-sizes, larval survival) are estimated from experimental data. The model predicts a male-biased sex allocation, which becomes more extreme as the probability of superparasitism (hosts parasitized more than once) increases. To test this prediction, we reared adult parasitoids at either low or high density, mated them, and presented them with unlimited hosts. As predicted, wasps produced more sons than daughters in all treatments. Males reared at high density (a potential cue for superparasitism) produced a higher male bias in their offspring than low-density males. Unexpectedly, female density did not affect offspring sex ratios. We discuss possible mechanisms for paternal control over offspring sex.

## Introduction

Sex allocation theory exemplifies the importance of frequency-dependent selection in population ecology. The theory successfully explains the 1∶1 sex ratio observed in many species, by positing a fitness advantage to the rarer sex. At equal reproductive value of male function and female function in the population, both sexes have identical fitness and equilibrium is reached (reviewed in [1], [2]). Extensions of the basic model predict the ecological conditions that select for “extraordinary sex ratios” [3]. Such circumstances include mating before dispersal among the offspring of a small number of females (Local Mate Competition), which favors the evolution of a female-biased sex allocation [4]. Deviations from equal sex allocation also occur under unequal competitive ability of males and females, which favors over-production of the weaker competitors [5]. Models that predict the effects of life-history features on sex allocation are often analytical, and focus on predicting the steady-state sex ratios rather than the dynamics leading to them [1].

Parasitoid wasps, hymenopterans whose larvae feed on the tissues of an arthropod host and eventually kill it, have a haplo-diploid sex determination system. That is, diploid females develop from fertilized eggs, while haploid males develop from unfertilized ones. This provides parasitoids with much flexibility in sex allocation, which is reflected in sex ratio shifts in response to various environmental conditions. For example, parasitoid sex ratios are affected by the presence of mated conspecifics [6], the sex of eggs laid by conspecifics [7], host size [8], [9], host age [10] and previous parasitism of the host [11]. In addition, maternal investment in parasitoids is restricted to the production of eggs and choice of a host for oviposition, thus resource allocation towards producing sons and daughters is similar and minimal. The proportion of sons at egg-laying (the primary sex ratio) therefore approximates the allotment of resources to male production (sex allocation). These characteristics make parasitoids popular models for testing predictions of sex allocation models. However, to make testable predictions, models need to incorporate the often complex life-history features of the parasitoids. These features include details on the mating systems, body sizes, developmental rates and competitive abilities of males and females, spatial distribution of hosts and dispersal abilities of adult parasitoids. It is often difficult to introduce this biological realism into standard analytical models of sex allocation. It is no less challenging to generate testable predictions from the models, and to subject them to experimental examination.

In the present study we aim to harness individual-based evolutionary simulation modeling to address these difficulties. We model sex allocation in the parasitoid *Copidosoma koehleri* (Encyrtidae: Hymenoptera) as a test case, because the unique life-history of this parasitoid challenges analytical sex allocation modeling. *C. koehleri* is a koinobiont egg-larval parasitoid of the potato tuber moth, *Phthorimaea operculella* (Gelechiidae: Lepidoptera). One egg is generally laid per ovipositor insertion into a host egg, but hosts often receive an additional egg during a later oviposition, thus becoming super-parasitized [12]. Eggs that develop within super-parasitized hosts may be of one sex or of different sexes. The eggs develop polyembryonically within the host, i.e. cleave repeatedly after oviposition to form clones of same-sex genetically identical wasps. Female clones contain more individuals, on average, than male clones (mean±SD clone sizes: 45.7±10.9 for females, 32.4±10.4 for males, [13]). Body size correlates negatively with the number of individuals in a brood (the total number of wasps emerging from a host, [14]). Female clones contain a morphologically distinct soldier larva that develops precociously, attacks competitors and dies before emerging from the host [15]. A high proportion of hosts contain two or more clones [16], which are usually mothered by different females [12], [17]. In hosts that contain both a male and a female clone, the female soldier eliminates some of the males, reducing the proportion of emerging males in the brood [16]. The duration of development is similar for males and females, thus both sexes emerge together in mixed-sex broods. Females are sexually receptive immediately after emergence, and often mate with male brood-mates in mixed-sex broods in the laboratory (pers. obs.). The extent of local mating in natural populations is unknown, but was shown to be high in the related species *C. floridanum* [18]. These intricacies complicate the prediction of ESS sex ratios in *Copidosoma* and other polyembryonic parasitoids, to the extent that “the development of specific theory is required” [2].

Qualitative considerations suggest that sex allocation in *C. koehleri* should balance between conflicting selective pressures: Equal reproductive value of male function and female function in large populations should result in equal sex allocation. However, males form smaller clones than females, and are outcompeted by them in mixed-sex broods. This generates a potential for Local Resource Competition between male and female larvae that develop within the same host. Such competition may favor an increased parental allocation towards production of the weaker competitor, that is, a male-biased primary sex ratio [5]. On the other hand, mating before dispersal may select for the production of excess females (a female-biased primary sex ratio) because of Local Mate Competition [3]. The evolved parental strategy regarding sex allocation should reflect the relative importance of these selective factors.

We predicted *C. koehleri*’s sex allocation using a simulation model, which allowed us to capture the details of the wasps’ life history and to follow the evolutionary dynamics of sex ratios in the simulated populations. The general simulation approach follows Lewis et al. [19], who used an individual-based model, evolving according to a Genetic Algorithm, to study factors that constrain optimal sex allocation in parasitoids. Lewis et al. [19] assumed that parasitoid females select the sex of each offspring according to host size, and modeled the assessment of host sizes using Artificial Neural Networks. In contrast, our model does not include an Aritificial Neural Network component. Instead, sex allocation is a genetically determined trait that is not affected by the parasitoids’ host encounter experience. We tested the main prediction generated by the model in a laboratory experiment.

## Methods

### Modeling

We set up an individual-based model, implemented in Java, to simulate the population dynamics of hosts and wasps for hundreds of generations, and to track the evolution of sex allocation in the parasitoid population. The model description follows the ODD (Overview, Design concepts, Details) protocol [20], [21]:

#### Purpose

JWasp is an agent-based discrete time simulation model designed to explore the behavior of *Copidosoma* parasitoid wasps, having the capability of choosing the sex of their offspring. Since it is unclear on what basis the wasps perform the sex choice, the model has been designed to evolve strategies according to different criteria, based on a random set of starting strategies.

#### State variables and scales

The model consists of three intuitive components. These are the environment, host and wasp models. The environment model holds three sets, which contain all entities in the simulation (Table 1). The Wasp model represents the individual wasps (the agents, Table 2). A Strategy is described using the variables of Table 3.

**Table 1:** The environment component of the simulation model.

Variable name	Brief description
Female wasps	Set of female wasps in simulation
Hosts	Set of hosts in simulation
Male wasps	Set of male wasps in simulation

Variables are listed in alphabetical order.

**Table 2:** The environment component of the simulation model.

Variable name	Brief description
Eggs laid	Counts the number of eggs produced by a female
Female genes	ID of mother
Females fertilized	The number of females fertilized by a male
Generations	Number of generations to simulate
ID	Unique ID
Lifespan	The lifespan of a wasp in number of eggs it can lay
Male genes	ID of male that fertilized a female
Sex	Boolean indicating the sex
Strategy	Strategy description
Virgin	Boolean indicating virginity of a female
Virgin’s son	Boolean indicating whether a male is a virgin’s son

Variables are listed in alphabetical order.

**Table 3:** The strategy component of the model.

Variable name	Brief description
Egg count influence	Influence of the number of eggs in a host on the probability to lay an additional egg
Host count	Number of hosts in simulation
Host limit	Carrying capacity of the host
Initial sex distribution	Initial proportion of males
Initial virgin ratio	Initial ratio of virgins
Initial wasp count	Initial number of wasps introduced into the simulation
In-host mating ratio	Proportion of wasps mating on the host, before dispersal
Sex choice distributions	The probability to lay a male egg. There is a value for empty hosts, and for hosts containing each combination of sex (male, female, mixed) and relatedness (related, unrelated, mixed)
Strategy inheritance	Proportion of male genes inherited by daughters
Survival distributions	For each possible host allocation, the number of wasps developing from a given egg is sampled from a normal distribution. We defined distribution parameters for male and female single-sex broods, and for male and female wasps within related and unrelated mixed-sex broods
Virgin son virility	Number of females that a virgin’s son can fertilize
Virility	Number of females that a mated female’s son can fertilize

Variables are listed in alphabetical order.

#### Process overview and scheduling

The model is based on the following list of rules:

- Female wasps lay eggs into hosts. The hatched larvae feed on the host and eventually kill it. After maturing into adults, they disperse.
- Generations do not overlap (i.e., parents are dead before the offspring disperse).
- Unfertilized virgin females can only produce male eggs.
- Fertilized females can freely choose the sex of their eggs.
- Sons of virgins have lower virility than sons of fertilized females.
- A fraction of the wasps can mate on the host, before dispersal.
- Some “soldier larvae” develop from female eggs. They kill unrelated competing larvae within the same host.
- An excess of eggs within a host will prematurely kill it and all other contained eggs.

Since generations do not overlap, each generation is initialized with a new set of hosts. The simulation allows each wasp to lay an egg at each time step, where the number of steps is the life span of the wasp. Furthermore, depending on its virility, each male can mate with one or several virgin females. After all time steps are completed the eggs proliferate, and the wasps’ offspring develop, and possibly mate, inside the host according to their respective survival distribution. Their daughters receive a strategy that is a linear combination of the parents’ strategies according to the “Strategy inheritance” parameter. Sons inherit their mothers’ strategies.

#### Design concepts - Basic principles

The simulation model is individual-based, and does not have a spatial component. It tracks large populations of parasitoids and their hosts. The initial composition of these populations can include individuals with several life-history strategies. The frequencies of the strategies may change over the course of the simulation due to selection and recombination, until a single stable strategy evolves. The modl’s parameter values can either be fixed throughout the simulation, or be allowed to evolve. In the present work we allowed the sex allocation random variable to evolve, and kept the remaining components of host and wasp strategies constant. Fixed parameter values were estimated from previous laboratory studies of *P. operculella*’s and *C. koehleri*’s life-histories [14]–[17], and are listed in Table S1.

#### Design concepts – Emergence

The emergent outputs of the model are the primary and secondary sex ratios of the wasp population, that is, the proportion of male eggs laid by a female and the proportion of adult males in the population. We examined how these sex ratios are affected by the wasps’ mating system and by the risk of intra-specific competition due to superparasitism.

#### Design concepts – Adaptation

The wasps base their sex choice on the state of the host encountered. The larvae developing inside the host interact with other larvae by competing for resources (exploitation competition) and through possible aggression of “soldiers” (interference competition). Females outcompete males, hence the adaptive benefit of producing females should depend on the extent of competition for host resources. As in other sex allocation models, the adaptive benefit from each sex is also negatively frequency-dependent, i.e. production of the rare sex should be advantageous. In addition, the wasps’ degree of mating before dispersal may affect the adaptive benefit of male vs. female production according to Local Mate Competition theory. The model predicts the combined effect of these, possibly conflicting, selective forces on the evolution of sex ratios.

#### Design concepts – Objectives

The male wasps’ main goal is to fertilize a maximal number of females. The females’ objective is to distribute their available eggs in a manner that would maximize their offspring survival. For the evolutionary development, the long term average size of the population is used as a fitness measure for comparing between strategies.

#### Design concepts – Learning

The model incorporates no learning. The wasps’ behavior is genetically determined.

#### Design concepts – Prediction

Agents do not predict the future, they just act based on the rules matching the current situation. The success of their behavior is evaluated through their reproductive performance, using a natural-selection-like procedure.

#### Design concepts – Sensing

Female wasps can sense the content of a host to some degree when choosing the sex of their next egg. There are three different possible information levels: (a) No information: the female only distinguishes empty hosts from parasitized ones. (b) Information on relatedness: the wasp also senses whether a parasitized host contains genetically related competitors. (c) Information on sex: the wasp senses the relatedness and sex of the competitors.

#### Design concepts – Interaction

Males and females interact only by mating and by competing inside a host. Females compete with each other through their egg-laying behavior. When encountering a host they react to the history of that host.

#### Design concepts – Stochasticity

Hosts are presented to wasps randomly. Furthermore, the wasps’ sex choices and survival rates are random variables, sampled from the respective distributions.

#### Design concepts – Collectives

The agents are not grouped and related wasps do not work together directly. Related embryos inside a host do form a collective, though, as they do not attack each other.

#### Design concepts – Observation

Data are extracted from the model by observing the number of male and female wasps, the population size, the occupation of the hosts, the number of eggs and the number of premature host deaths. If multiple strategies are simulated, we observe the number of distinct strategies in the simulation, which will change in frequency due to recombination and selection, and will eventually converge to a single strategy.

#### Initialization

The model is initialized with a number of empty hosts and a randomly sampled wasp population according to the initialization parameters. Different strategies can be provided through the user interface. The initial number of wasps using each strategy is defined by the user.

#### Input data

After initialization the model does not take any additional input.

#### Submodels - Wasp model

Each female wasp is presented with a host at each time step. If the host passes the egg-laying criterion of the wasp it receives an egg. If a wasp is fertilized, it chooses the sex of the egg based on information about the host. An unfertilized female can only lay male eggs. The only action a male wasp can perform is to mate with a female.

#### Submodels - Host model

Hosts whose attractiveness exceeds the threshold are accepted for parasitism. An egg count influence parameter describes the hosts’ decrease in attractiveness after having been parasitized by a wasp egg. Parasitized hosts that are above threshold may potentially be superparasitized, either by the same female or by a different one. If the host gets too crowded it dies prematurely. Freshly-emerged wasps may mate on the host. Eventually the host will die and the wasps will disperse as the next generation.

**Figure 1:**
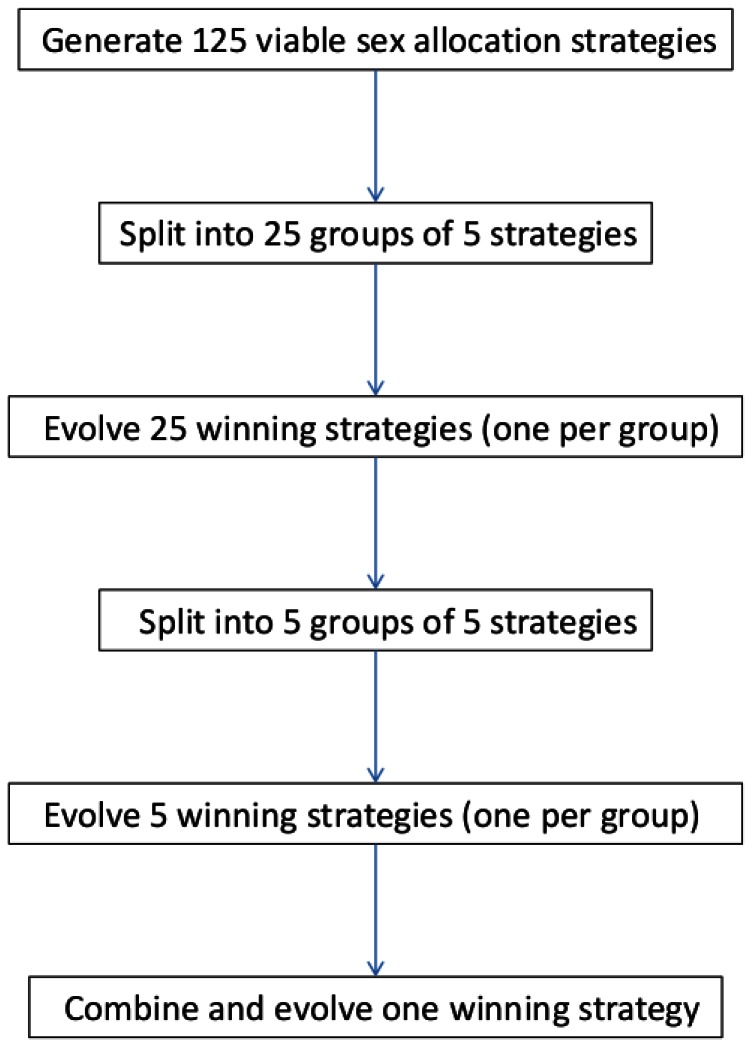
Steps in the evolution and selection of the wasps’ sex allocation strategy. This protocol was applied to each of the modeled scenarios and host acceptance thresholds.

To search for evolutionarily stable sex allocations, we started with 125 random viable strategies (sex allocation values that ranged 0–1) for each of three simulated scenarios, which are described in the next paragraph. “Viable” means that a population consisting of individuals using such allocation exclusively would not go extinct. We split this set into 25 subsets of five strategies each. Each strategy was initially represented by 100 wasp females. In a first round, each subset was used to simulate the dynamics of the competing sex allocation strategies. Successful strategies eventually took over the population by producing more offspring, which inherited their parents’ strategies, compared to less successful sex allocation strategies. In a second round, the 25 winners of the first round were again randomly split into five subsets, and were allowed to compete to produce five winning strategies. Finally, the most successful strategy among these five was determined in a third round of simulations. This evolution and selection protocol is summarized schematically in Fig. 1. It was replicated five times for each set of conditions. We report on the mean±SD winning sex allocation in each set of replicates.

We first simulated a “symmetric scenario”, which included equal embryonic proliferation and survival of male and female larvae, and mating that occurs before dispersal, i.e. a mating structure that promotes Local Mate Competition. Under this scenario, males and females have equal developmental prospects, therefore a female-biased sex allocation is expected to evolve. Next, we simulated two “asymmetric scenarios”, where females proliferate more than males and outcompete them in superparasitized hosts. In one set of simulations, the wasps mate after dispersal, while in a second set they mate with individuals from the same host (if available) immediately after emerging from the host. The comparison between the “symmetric” and “asymmetric” scenarios should reveal the selective effect of the females’ competitive advantage on sex allocation. We further expected the comparison between the two “asymmetric” scenarios to reflect the effect of mating structure on the predicted sex ratio. Each of the three scenarios was simulated for nine host acceptance thresholds. Low host acceptance thresholds in our simulation model are equivalent to high levels of superparasitism, as already-parasitized hosts are accepted by wasps and receive an additional parasitoid egg. Therefore, by varying host acceptance thresholds we were able to predict the evolved sex allocations under rising risk of superparasitism, leading to increasing levels of between-clone competition.

### Experiment

#### Insects and laboratory conditions

Laboratory-reared hosts and parasitoids were used for the experiments. Their rearing followed a standard protocol [22]. The temperature during rearing and throughout the experiment was 27±2°C, and relative humidity was ∼60%. Hosts used for the experiment were 0- to 24-h-old moth eggs. Wasps were 0- to 24-h old males and females from single-sex broods.

#### Experimental design

The experiment was designed to test the simulations’ main predictions (see Results below). Specifically, we aimed to examine whether sex allocation in *C. koehleri* deviates from equality, and whether it is affected by the risk for superparasitism. We reared the wasps at either high or low density before exposing them to hosts, to generate an expectation of either high or low risk of superparasitism. This manipulation follows Shuker et al. [23], who found that the rearing density of adult wasps affected sex allocation in *Nasonia* parasitoids. 30 female-only and 30 male-only broods were used for the experiment. Upon emergence from the host, 20 wasps from each brood were moved to a 13×100 mm (diameter×length) test tube for 48 hours. These wasps formed the high-density (HD) treatment. Eight additional wasps from the same brood were housed in pairs in four additional test tubes of the same dimensions, and formed the low-density (LD) treatment. The wasps were supplied with honey as a food source, but not with hosts, during this time. We then allowed wasps from all four density combinations to mate (LD♂×LD♀, LD♂×HD♀, HD♂×LD♀, HD♂×HD♀). For each mating combination, we placed two males and two females in a petri dish for 6 hours. This was done in two replicates, i.e., 16 individuals (2/dish×2 replicates×4 treatments) from each brood. Thus, total sample size was 2 replicates×4 mating combinations×30 parental clones = 240. Mating normally occurs within 5 minutes of encounter between males and females in *C. koehleri*, and lasts for a few seconds. Females copulate repeatedly when housed with males (pers. obs.). We therefore expected the females to be mated after 6 hours of cohabitation with males. >50 hosts (moth eggs), haphazardly selected from the insectary culture, were then introduced into each dish, and the wasps were allowed to parasitize them for 24 hours. As *C. koehleri* females parasitize ca. 20 hosts during their first day after emergence [24], the number of hosts offered in the experiment did not limit the wasps’ fecundity. The distribution of host sizes is not expected to differ among the four experimental treatments. Thus, any potential effects of host size on sex allocation should be similar across treatments. Hosts were reared on potatoes until parasitoid pupation. Each host mummy, containing a brood of wasp pupae, was then transferred into an individual test tube. Parasitoids were sexed after emerging from the hosts. Parasitoid brood sizes were determined in a subsample of 150 broods, collected from 15 replicates from each treatment.

#### Experimental data analysis

Two-way ANOVAs were used to analyze the effect of maternal and paternal rearing density on the proportions of male, female and mixed-sex broods. Proportions were arcsine-transformed prior to analysis. We used the proportion of mixed-sex broods (0.35) as an estimate for the frequency of superparasitized hosts in the whole dataset. Thus, we assumed that 0.35 of the all-male and of the all-female broods resulted from two eggs of the same sex, and that all mixed-sex broods developed from one male and one female egg. Based on these assumptions, we calculated the estimated proportion of male eggs (primary sex ratio) produced in each of the treatments.

Males comprise ca. 1/3 of mixed-sex broods [14]. Therefore we estimated the proportion of males in each treatment (the secondary sex ratio) as (total # wasps in all-male broods+1/3 of the # wasps in mixed-sex broods)/total # wasps.

## Results

### Simulation Model

**Figure 2:**
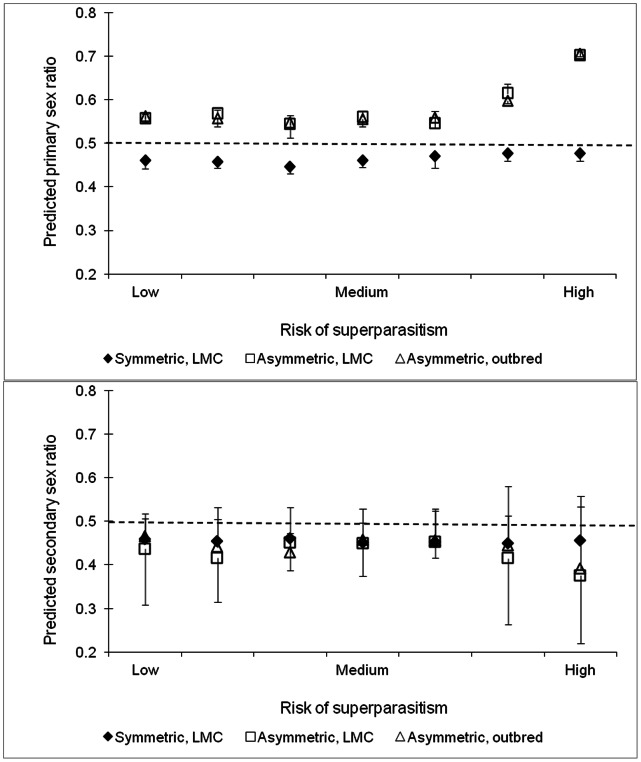
Evolved primary (top) and secondary (bottom) sex ratios as a function of superparasitism level (modeled by varying host acceptance thresholds). ♦ – assuming equal numbers of emerging males and females per clone, and mating before dispersal. □ – assuming developmental advantage to females and mating before dispersal. Δ – assuming developmental advantage to females and dispersal before mating. The simulations crashed at the lowest simulated value of risk of superparasitism because of extinction of the parasitoid population. They also collapsed at the highest superparasitism risk level, because the heavily superparasitized hosts died prematurely. The graph therefore shows the seven superparasitism values that allowed stable population dynamics. The dashed line marks the 1∶1 sex allocation.

The simulation model predicts the evolution of a female-biased primary sex ratio when male and female wasps have similar developmental prospects, and mating occurs before dispersal (the “symmetric scenario”). This prediction is not affected by the risk of superparasitism (Fig. 2, top). Secondary sex ratios are also female-biased, at all levels of superparasitism (Fig. 2, bottom). In the two scenarios with competitive asymmetry between the sexes, an excess of male-egg production is predicted. The male bias in the primary sex ratio is predicted to increase with higher risk of superparasitism, but is unaffected by the parasitoids’ mating structure (Fig. 2, top). However, the predicted proportion of males in the population (secondary sex ratio) is lower than 0.5, and decreases further at high levels of superparasitism (Fig. 2, bottom).

### Laboratory Experiment

The experiment manipulated the effect of wasp rearing density, a possible cue for the risk of superparasitism. We expected wasps reared at high density to anticipate more superparasitism in their hosts than wasps reared at low density. According to the model’s prediction, we expected that wasps in all treatments would produce a male-biased primary sex ratio, but that the bias would be more extreme in the high-density treatments than in the low-density treatments. We further expected primary sex ratios to be influenced by maternal rearing densities if sex allocation is controlled by females, and by paternal rearing densities if sex allocation is under male control. If both males and females influence sex allocation in *C. koehleri*, then primary sex ratios are expected to be highest when both parents are maintained at high density, lowest when both parents are kept at high density, and intermediate for the remaining two treatments.

**Figure 3:**
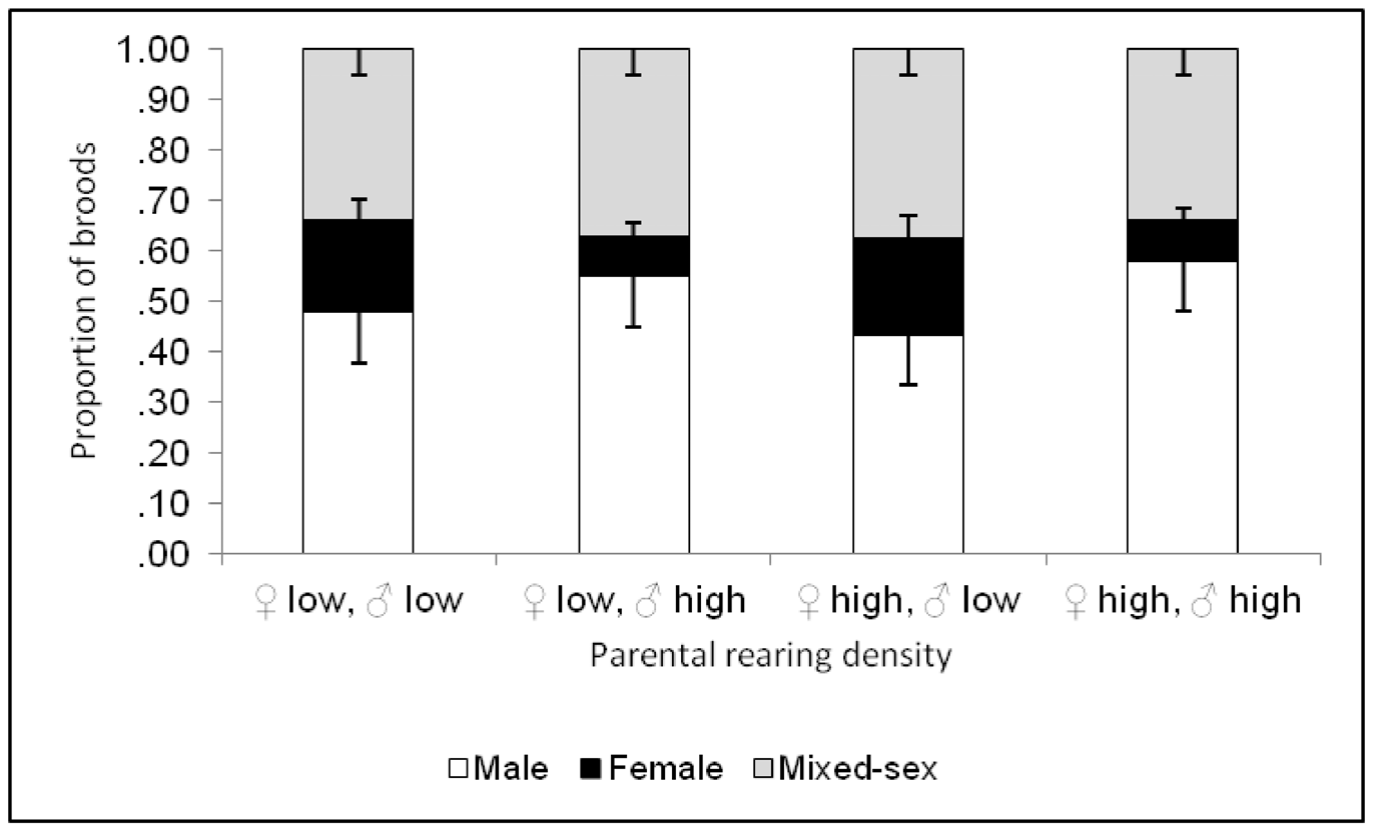
Mean (and associated SD) proportions of male-only, female-only and mixed-sex broods, produced by parents that were housed at high vs. low density for 48 hours after emergence.

The frequencies of male, female and mixed-sex (both male and female) broods are reported in Fig. 3. The proportions of male-only broods were significantly affected by paternal rearing density, but not by maternal density (two-way ANOVA on arcsine-transformed data: F_3, 189_ = 2.077, P = 0.10 for the complete model; F_1, 189_ = 1.94, P = 0.018 for paternal density; F_1, 189_ = 0.04, P = 0.841 for maternal density). Similarly, the proportions of female-only broods were significantly influenced by paternal densities only (F_3, 189_ = 3.345, P = 0.02 for the complete model; F_1, 189_ = 9.932, P = 0.002 for paternal density; F_1, 189_ = 0.044, P = 0.834 for maternal density). Interactions between maternal and paternal densities were non-significant. The proportions of mixed-sex broods were not significantly affected by parental rearing conditions. In conclusion, all-male broods increased in frequency, at the expense of all-female broods, in the two treatments with high paternal density.

**Figure 4:**
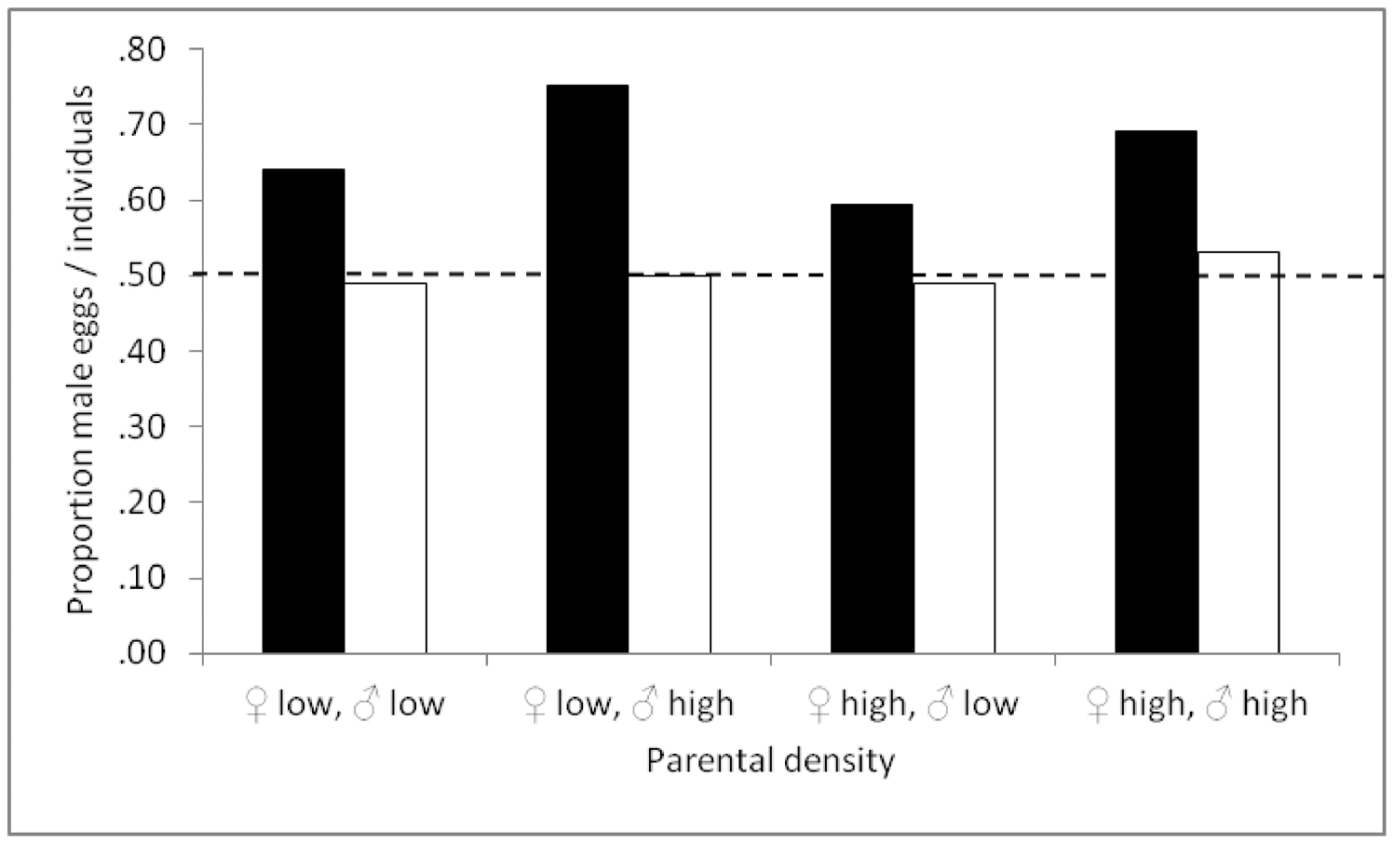
Estimated proportions of male eggs (primary sex ratio, black bars), and proportions of male individuals (empty bars) produced by parents that were housed at high vs. low density for 48 hours after emergence. The dashed line indicates the 1∶1 sex ratio.

The occurrence of mixed-sex broods in ca. 35% of the hosts indicates that they were parasitized more than once. In *C. koehleri*, superparasitism occurs in all sex combinations: male-male, female-female and female-male [16]. We estimated the proportions of male eggs laid in the whole dataset, by conservatively assuming that 35% of the single-sex hosts were parasitized by two wasp eggs as well. This provides an estimate of the primary sex ratio in the four density treatments (Fig. 4, black bars). In line with our working hypothesis, this ratio is male-biased in all treatments. The bias is especially pronounced in the treatments of high male density, consistent with a paternal influence on sex allocation.

Wasps from all treatments were provided with hosts without restriction. This allowed us to study the effects of parental sex allocation on secondary sex ratios without possible confounding effects from varying competition levels among developing offspring. Indeed, the proportion of mixed-sex broods (an estimate for the level of superparasitism) did not vary significantly across treatments. This suggests that manipulation of wasp rearing density affected the parent’s anticipation of superparasitism, but not the degree of superparasitism experienced by their offspring. The secondary sex ratios (proportion of adult male wasps) in all treatments were close to 0.5 (Fig. 4, empty bars), in spite of the much higher estimated proportion of male eggs laid.

## Discussion

Polyembryonic parasitoids provide prime examples of within-family conflicts over sex allocation, because the evolutionarily stable sex ratios may differ from the perspectives of different family members [2]. Sons and daughters are more related to their clone-mates (which share their sex) than to other siblings, while mothers are equally related to their sons and daughters. Thus, daughters benefit from lower sex ratios than sons in many situations, while mothers favor intermediate sex ratios. Gardner et al. [25] suggested that this conflict is resolved in favor of the daughters, through the evolution of the female soldiers that kill male sibs and thereby reduce the secondary sex ratio. Our model and experimental data suggest that, at least in *C. koehleri*, the soldiers’ aggression is counteracted by a male-biased primary sex ratio determined by the parents. Thus, our experiment points at the potential of the parents to influence offspring sex allocation in *C. koehleri*, consistent with their putative role in this evolutionary conflict.

In line with standard sex allocation theory, our simulation predicts a female-biased primary sex allocation under LMC and equal survival/proliferation of both sexes (Fig. 2, top, “symmetric scenario”), at all levels of superparasitism. The predicted secondary sex ratio remains female-biased (Fig. 2, bottom, “symmetric scenario”). The introduction of advantages in proliferation and competition to female larvae selects for a male-biased primary sex ratio, whether mating occurs before or after dispersal. This implies that developmental asymmetry drives the evolution of sex allocation more strongly than mating structure in our system. In *Nasonia* parasitoids, on the other hand, LMC is predicted to affect sex allocation much more strongly than the competitive asymmetries between male and female larvae [5]. A possible reason for these different findings is that the female soldier caste of *C. koehleri* generates a powerful mechanism of sex ratio regulation through larval competition, which is absent from monoembryonic species. Increasing the risk of superparasitism selects for even more male-biased primary sex ratios in our model. This may compensate for the greater proportion of male larvae killed by female soldiers. In both “asymmetrical scenarios”, secondary sex ratios are lower than 0.5 in spite of the male-biased primary sex allocation (Fig. 2, bottom), due to the female competitive advantage. The lowest secondary sex ratios are predicted at the highest levels of superparasitism, when female soldiers most frequently encounter and kill male competitors within the host.

The experimental results are consistent with the main predictions of the model, in that primary sex ratios were male biased in all treatments. A similar excess of male broods was also found in a field-collected sample of hosts parasitized by *C. koehleri* [16]. Also in line with the model, an environmental signal for increased risk of superparasitism (high rearing density) increased the male bias even further. By setting up all four parental density combinations, we learned that this increase in sex ratio was in response to high rearing density of fathers, but not of mothers. An alternative interpretation for the elevated sex ratio is that high parasitoid density serves as a signal for reduced future local mate competition (because it predicts more founding wasps per host), rather than increased future competition for host resources [5]. An additional interpretation is that females use increased wasp densities as a signal of better prospects of finding mates, and thus a higher potential for producing daughters in the next generation. Because of negative frequency dependence, this reduces the fitness benefit from producing daughters, favoring a higher proportion of males among the offspring of mated females [26]. These two alternative interpretations assume mating before dispersal, while our model predicts little effect of mating structure on sex allocation in *C. koehleri*.

Past studies have traditionally stressed the role of mothers in mediating offspring sex allocation, because of their greater involvement in parental care. Such control can involve fertilization (e.g., not using sperm of some mates [27], [28]), developmental conditions that benefit embryos of one sex [29] or food provisioning to young that favors one of the sexes [30]. However, evidence for the ability of males to also affect their offspring’s sex is gradually accumulating [31], as exemplified in a few studies on hymenopterans: in the parasitoid *Encarsia*, females develop on ‘normal’ (whitefly) hosts, whereas males can hyperparasitize and kill the female larvae of their own species, thereby reducing the proportion of females [32]. In *Nasonia*, males from different strains sire offspring that vary in sex ratios [28]. Mate-guarding after copulation by male *Urolepis rufipes* parasitoids increases the proportion of daughters among the offspring [33]. Similarly, nest-guarding by males in the mud-daubing wasp *Trypoxylon politum* allows their mates more time for nest provisioning, a possible strategy for enhancing the production of daughters [34].

By what mechanism might male rearing density have affected offspring sex ratios in the present experiment? A possible proximate mechanism involves parental epigenetic control over sex allocation. Such control has been demonstrated in *Nasonia vitripennis*, where maternal input of transformer (Nvtra) messenger RNA is required for female development from fertilized eggs [35]. A major pathway of epigenetic control involves regulation of DNA expression levels through gene methylations. Methylations, as well as homologues to vertebrate genes that control them, are documented in a wide range of hymenopterans. This provides a potential molecular mechanism whereby parental environment could affect offspring sex ratios and resolve kin conficts [36]. Thus, males may potentially affect offspring sex allocation through substances transmitted in their ejaculates during copulation, possibly counteracting female control over fertilization.

Hymenopteran males transmit their genes through daughters only. Thus, if males can indeed influence offspring sex, they would be expected to favor female production under all circumstances. This prediction does not agree with our finding that offspring primary sex ratios varied with paternal rearing densities. A possible interpretation is that males kept at high density were less successful in mating or fertilizing their mates than low-density males, leading to a higher proportion of haploid, male offspring. This interpretation is non-adaptive and considers high-density rearing as a constraint on male fitness. Another possible non-adaptive pathway could involve paternally-transmitted selfish genetic elements that act as sex-ratio modulators. In the parasitoids *Trichogramma* and *Nasonia*, a nuclear extra chromosome called PSR is carried by males only. PSR turns eggs destined to develop as females into males, enhancing its own transmission into the offspring generation [37]. Such modulation of sex ratios could account for our experimental results, if the extent of modulation depends on male rearing density.

To conclude, our study demonstrates the power of individual-based evolutionary modeling in exploring frequency-dependent traits in organisms, and in generating testable hypotheses about them. The simulation approach is particularly useful for species with complex life-histories, which commonly challenge analytical modeling. While we applied the model to simulate the evolution of sex allocation in polyembryonic parasitoids, the individual-based approach may be fruitfully applied to additional life-history traits and organisms as well. Male rearing densities affected offspring sex allocation in our experiment. The mechanism and evolutionary significance underlying this finding still need to be elucidated.

## Supporting Information

Table S1 **Parameter values for the simulation model.**
(DOC)
