# Supplementary material for: Sex Allocation in a Polyembryonic Parasitoid with Female Soldiers: An Evolutionary Simulation and an Experimental Test
Source: PLoS One. 2013 Jun 3;8(6):e64780. doi: 10.1371/journal.pone.0064780 (PMC3670893; doi:10.1371/journal.pone.0064780)
Supplement: Table S1 — Parameter values for the simulation model. (DOC) [file pone.0064780.s001.doc]

| Parameter | Initial parameter value | Parameter value fixed? |
| --- | --- | --- |
| Number of hosts | 500 | No |
| Number of simulated generations | 2000 | Yes |
| Number of wasps per strategy | 100 | No |
| Wasp lifespan (arbitrary time units) | 20 | Yes |
| Number of females fertilized by a male | 1 | Yes |
| Frequency of LMC | 0, 1 | Yes |
| Quality of non-parasitized host | 0.6 | Yes |
| Threshold for host acceptance | 0.25-0.6 | Yes |
| Reduction of host quality with each parasitoid egg | 0.3 | Yes |
| Wasp information state (none, identifies self-parasitized hosts, identifies sex of eggs in previously parasitized hosts) | None | Yes |
| Sex allocation | 0.5 | No |
| Probability of inheriting maternal sex allocation strategy | 0.6 | Yes |
| Sex allocation inheritance mode (average of the parental allocations or allocation of one of the parents) | Average allocation | Yes |
| Proportion of virgin wasps | 0.1 | Yes |
| Maximal number of wasps / brood | 100 | No |
| No. individuals per clone in different types of broods, symmetric scenario | 40.0±10.0 Single male (M) or single female (F)  20.0±10.0 M or F in superparasitized hosts |  |
| No. individuals per clone in different types of broods, asymmetric scenario | 32.7± 11.0 Single M  45.8± 10.9 Single F  24.0±10.0 MM  30.0±10.0 FF  53.4±7.0 F in brother-sister broods  16.9±2.2 M in brother-sister broods  42.9±7.1 F in unrelated MF broods  13.6±2.6 M in unrelated MF broods | Yes |
